# Characterization of microRNA profiles in the mammary gland tissue of dairy goats at the late lactation, dry period and late gestation stages

**DOI:** 10.1371/journal.pone.0234427

**Published:** 2020-06-08

**Authors:** Rong Xuan, Tianle Chao, Aili Wang, Fuhong Zhang, Ping Sun, Shuang Liu, Maosen Guo, Guizhi Wang, Zhibin Ji, Jianmin Wang, Ming Cheng

**Affiliations:** 1 Shandong Provincial Key Laboratory of Animal Biotechnology and Disease Control and Prevention, College of Animal Science and Veterinary Medicine, Shandong Agricultural University, Tai'an, Shandong Province, P.R. China; 2 Qingdao Research Institute of Husbandry and Veterinary, Qingdao, Shandong Province, P.R. China; University of Agricultural Sciences, INDIA

## Abstract

MicroRNAs (miRNAs) play an important role in regulating mammary gland development and lactation. We previously analyzed miRNA expression profiles in Laoshan dairy goat mammary glands at the early (20 d postpartum), peak (90 d postpartum) and late lactation (210 d postpartum) stages. To further enrich and clarify the miRNA expression profiles during the lactation physiological cycle, we sequenced miRNAs in the mammary gland tissues of Laoshan dairy goats at three newly selected stages: the late lactation (240 d postpartum), dry period (300 d postpartum) and late gestation (140 d after mating) stages. We obtained 4038 miRNAs and 385 important miRNA families, including mir-10, let-7 and mir-9. We also identified 754 differentially expressed miRNAs in the mammary gland tissue at the 3 different stages and 6 groups of miRNA clusters that had unique expression patterns. Gene Ontology (GO) and Kyoto Encyclopedia of Genes and Genomes (KEGG) analyses showed that GO terms such as mammary gland development (GO:0030879) and mammary gland morphogenesis (GO:0060443) and important signaling pathways, including the insulin signaling pathway (chx04910), hippo signaling pathway (chx04390) and estrogen signaling pathway (chx04915), were enriched. We screened miRNAs and potential target genes that may be involved in the regulation of lactation, mammary gland growth and differentiation, cell apoptosis, and substance transport and synthesis and detected the expression patterns of important genes at the three stages. These miRNAs and critical target genes may be important factors for mammary gland development and lactation regulation and potentially valuable molecular markers, which may provide a theoretical reference for further investigation of mammary gland physiology.

## Introduction

The mammary gland of dairy goats is an economically important organ because of its milk production, and lactation performance is closely related to the stage and status of the mammary gland [1]. During the reproductive cycle of dairy goats, the mammary gland undergoes repetitive processes, including repeated growth, as well as functional differentiation and degeneration, with each dynamic process accompanied by the proliferation and apoptosis of mammary epithelial cells [2]. Milk secretion is affected by the number of mammary secretory cells, the secretory ability and activity of mammary epithelial cells and the transport capacity of the vascular system [3]. The developmental characteristics at different physiological stages of the mammary gland make it an ideal model with which to study the regulation of lactation and signal transduction pathways related to cell proliferation, differentiation and apoptosis [4].

Changes in the physiological and structural functions of the mammary gland at different developmental stages are usually closely related to various hormones, such as growth hormone, estrogen, prolactin, and progesterone; various growth factors, such as epidermal growth factor, transforming growth factor, and amphiregulin; and microRNAs (miRNAs) that regulate gene expression [5–8]. miRNAs are a class of small noncoding RNAs originally identified in *Caenorhabditis elegans* that are 18–25 nt in length and involved in the posttranscriptional regulation of gene expression in animals [9]. Some miRNAs can bind to multiple messenger RNA (mRNA) 3’ untranslated regions (UTRs) and thus have the capability of regulating a large number of genes [10]. The miRNAs of most mammals are involved in the regulation of multiple biological processes, including cell proliferation and apoptosis, tissue differentiation, material and energy metabolism and hormone secretion [11]. Additionally, miRNAs also play important roles in mammary gland development and lactation regulation. For example, miR-31 promotes mammary epithelial cell proliferation and inhibits mammary epithelial cell differentiation [12]. Deregulation of miR-30b leads to impaired mammary gland structure and function during lactation and involution [13]. In recent years, significant progress has been made in miRNA studies, as shown by the number of mature miRNAs available in the miRBase database (release 22.1). A total of 2656, 1978, 1030, 436 and 153 mature miRNAs have been collected for *Homo sapiens*, *Mus musculus*, *Bos taurus*, *Capra hircus*, and *Ovis aries*, respectively. Many studies have been carried out in mice and humans, but research on ruminants is still relatively rare. Therefore, the identification, expression pattern analysis and functional analysis of miRNAs in the mammary glands of dairy goats could provide a deeper understanding of the posttranscriptional mechanisms of mammary gland development and lactation regulation, which would lay a theoretical foundation for revealing the mechanism by which miRNAs participate in mammary gland development and lactation regulation.

From the onset of lactation to the peak period, the number and secretory activity of mammary epithelial cells increase [14]. After the peak of lactation, milk yield begins to decrease gradually, and the ability of mammary epithelial cells to synthesize proteins, fats, and sugars decreases [15]. Then, at the late lactation (LL) stage, the mammary gland undergoes a transition from lactation to nonlactation, mammary gland synthesis and milk secretion gradually stop, the lobular/acinar structure of the mammary gland disintegrates, a large amount of mammary gland tissue degrades, and a large number of mammary cells undergo apoptosis [16]. During the long lactation period, dairy goats consume a large amount of nutrients and become extremely fatigued. The dry period (DP) is an important and necessary stage for dairy goats, allowing body recovery, energy storage and physiological activity recovery [17]. The DP represents not only the cessation of milk synthesis and secretion but also the beginning of mammary tissue remodeling [18]. A previous study showed that during the DP, mammary gland cells continue to proliferate and differentiate to prepare for the next lactation cycle [19]. During the late gestation (LG) stage, the numbers of epithelial cells, ductal branches and developing acini increase rapidly due to the higher degree of differentiation and proliferation of mammary epithelial cells under the action of estrogen, growth hormone, prolactin and a variety of cell growth factors [20]. At this time, milk-secreting cells synthesize various proteins, sugars and fats and transport them to the extracellular acinar cavity for storage [21].

In recent years, RNA-seq has become a powerful tool for large-scale gene identification, mining and expression analysis, and it provides a reliable, qualitative and quantitative method for biological research on transcription. Previously, we used RNA-seq technology to perform high-throughput sequencing of mammary gland tissue of Laoshan dairy goats at the early (20 d postpartum), peak (90 d postpartum) and late (210 d postpartum) lactation stages, identify and functionally annotate the miRNAs in mammary gland tissue and map miRNA expression profiles relevant to the mammary gland biology of Laoshan dairy goats [22–24]. But analyses of the miRNA transcriptome in the mammary gland tissue of dairy goats at the LL, DP and LG stages are lacking. During LL, mammary gland tissue degenerates and the number of cells undergoing apoptosis greatly increases, while mammary gland tissue is remodeled during the DP. At the LG stage, mammary gland tissue exhibits a higher degree of differentiation, and the cell proliferation, substances transport, synthesis and lactation capacity of the tissue are significantly improved. Do miRNAs participate in the regulation of physiological processes such as mammary gland tissue differentiation, cell growth, milk synthesis and secretion during these three developmental stages? To answer this question, we sequenced small RNAs in the mammary gland tissue at different stages using Illumina/Solexa high-throughput sequencing technology and identified and analyzed miRNAs closely related to mammary gland development. Our results may enrich and clarify miRNA expression profiles in relation to mammary gland development and lactation regulation in dairy goats and provide a theoretical reference for understanding the mechanism of posttranscriptional regulation at different developmental and physiological stages.

## Materials and methods

### Ethics statement on experimental animals

All animal protocols used in this study were approved by the animal protection and ethics committee of Shandong Agricultural University (protocol number: SDAUA-2018-048), and best efforts were made to reduce animal suffering during the experiments.

### Mammary gland tissue collection and RNA isolation

Laoshan dairy goats at the LL stage (n = 3, 240 d postpartum), the DP stage (n = 3, 300 d postpartum) and the LG stage (n = 3, 140 d after mating) were used in this study. The goats were 4 years old on average (third parity) and were from the Qingdao Aote goat breeding farm (S1 Table). All goats had free access to food, were healthy and disease free and were bred and managed under the same conditions. After pentobarbital sodium (100 mg/kg) was injected into the jugular vein of the goats, the muscles relaxed, and the heart and respiration were arrested, we immediately dissected the goats and collected the mammary gland. Part of the mammary gland tissue was stored in 4% paraformaldehyde immediately, and the remaining tissue was placed in RNase-free cryotubes after rinsing with diethylpyrocarbonate (DEPC)-treated water and stored in liquid nitrogen. Total RNA was extracted using a TRIzol^™^ Reagent Kit (Invitrogen, Carlsbad, CA). RNA concentration and integrity were examined using an Agilent 2100 Bioanalyzer (Agilent Technologies, Waldbronn, Germany), and RNA samples with an RNA integrity number (RIN) > 8 were used for subsequent experiments.

### Small RNA library construction and high-throughput sequencing

Nine small RNA libraries were established using the total RNA isolated from mammary gland tissue at the LL, DP and LG stages (LL1, LL2, LL3, DP1, DP2, DP3, LG1, LG2 and LG3) with an NEBNext^®^ Ultra^™^ RNA Library Prep Kit (Illumina, San Diego, USA). For each small RNA library, 1 μg of total RNA was subjected to electrophoresis using 12% Tris-borate ethylenediaminetetraacetic acid (TBE)-urea polyacrylamide gel electrophoresis (PAGE) gels (Invitrogen) to obtain small RNA fragments with a length between 18 and 30 nt. The 3’ and 5’ ends of the RNA were ligated with T4 RNA ligase 2 and reverse transcribed to obtain cDNA based on the 3’ and 5’ adapters ligated to the small RNA. The target library was amplified using PCR, and dsDNA fragments between 140 and 160 bp in length were extracted using TBE gel electrophoresis. The libraries were subjected to sequencing using an Illumina/Solexa system after quality tests.

### Preprocessing and comparison of the sequencing data

The data were subjected to quality assessment using FastQC software. According to the quality assessment results, the sequencing adapters were processed with Cutadapt software to eliminate reads with 10% or more unknown bases and a length greater than 30 bp or less than 18 bp and to retain reads with a sequencing quality (Q) > 30. The filtered clean reads were subjected to sequence comparison with the Silva (https://www.arb-silva.de/), GtRNAdb (http://lowelab.ucsc.edu/GtRNAdb/), Rfam (http://rfam.xfam.org/) and Repbase (https://www.girinst.org/repbase/) databases using Bowtie software. Subsequently, the reads were annotated as repeat reads and noncoding RNAs (ncRNAs), including ribosomal RNAs (rRNAs), transfer RNAs (tRNAs), small nuclear RNAs (snRNAs) and other RNAs. The comparison results were summarized, and the short reads were annotated with classifications. Unannotated reads containing miRNAs were obtained after filtering ncRNAs and repeated sequences. The filtered unannotated reads were then aligned with the goat reference genome (ftp://ftp.ncbi.nlm.nih.gov/genomes/all/GCF/001/704/415/GCF_001704415.1_ARS1) to determine the locus information on the reference genome. Known miRNAs were identified, and novel miRNAs were predicted with miRDeep2 software [25] using the mature and hairpin sequences of goat, cow and human in the miRBase database (http://www.mirbase.org/). Transcripts per million (TPM) values of miRNA expression were calculated by miRDeep2 software. According to the TPM values, miRNAs were divided into four groups: the high-expression group (TPM ≥ 500), medium-expression group (500 > TPM ≥ 10), low-expression group (10 > TPM ≥ 1), and ultra-low-expression group (1 > TPM). The detected known miRNAs and novel miRNAs were subjected to family analysis based on sequence similarity to determine the conservation of miRNAs throughout evolution.

### Screening for differentially expressed miRNAs

Before analysis of differential expression, the data were assessed by RUVSeq (V3.8) software, and factors of unwanted variation (for instance, batch effects) were estimated using the replicate samples according to the RUV function [26]. Additionally, expression data for the 9 libraries were standardized. The degree of data variability was examined using principal component analysis (PCA) and relative logarithmic expression (RLE) analysis. The hypothesis of differential expression of miRNAs among the LL, DP and LG stages was tested using DESeq2 software [27]. The miRNAs at different developmental stages were considered to be differentially expressed when the false discovery rate (FDR) was ≤ 0.01 and the log2 fold change was ≥ 1. Z-score-normalized expression levels of the differentially expressed miRNAs were used to draw a heat map. At the same time, three sets of volcano plots of differentially expressed miRNAs were drawn, and miRNAs whose absolute log2 fold change value was greater than 1.5 and whose expression level was equal to or higher than the medium expression level were screened (TPM ≥ 10). The differentially expressed miRNAs whose expression levels were in the top 20 at different developmental stages (LL, DP, and LG) were screened and then displayed with bar graphs.

### Temporal pattern analysis of differentially expressed miRNAs

To further understand the expression pattern of differentially expressed miRNAs in mammary gland tissue during the 3 stages, we performed cluster analysis of the differentially expressed miRNAs with TCseq software (http://bioconductor.org/packages/release/bioc/html/TCseq.html) using the c-means method and drew line graphs of the expression levels of different miRNAs at the 3 developmental stages.

### miRNA target gene prediction and functional annotation analysis

Target genes of differentially expressed miRNAs were predicted using TargetScan [28] and miRanda software [29]. The overlapping results predicted from the software were used as the final miRNA targets. Gene Ontology (GO) functional analysis and Kyoto Encyclopedia of Genes and Genomes (KEGG) pathway analysis of the differentially expressed miRNAs were performed using the online software DAVID [30]. All genes were used as the background list, and target genes were selected as candidates from this list. P values were obtained by the hypergeometric distribution test and were subjected to Benjamini-Hochberg correction for multiple testing to obtain the FDR. GO annotation results and KEGG pathways with an FDR < 0.05 were selected. The GO annotation results for target genes in the three groups are displayed according to category (biological processes, cellular components, and molecular functions) and divided into 6 categories according to specific functions: apoptosis-related, hormone-related, lactation-related, mammary gland-related, metabolism-related, and transport-related. Based on the KEGG annotations, we identified 4 categories. The GO and KEGG results were displayed by the R software (V3.6.2).

### Construction of miRNA and target gene regulatory networks

Based on the GO and KEGG analysis results, potential target genes and corresponding miRNAs related to basic substance transport and synthesis, mammary gland development, cell growth and differentiation, lactation regulation, and apoptosis were selected. The regulatory networks of differentially expressed miRNAs and target genes were drawn using Cytoscape (v37.1) [31]. The regulatory networks were analyzed using the NetworkAnalyzer plug-in in Cytoscape, and the degree score of each node was calculated. The screening condition for hub miRNAs and hub genes was a degree score ≥ 7. Heat maps and chord diagrams were drawn in R software to show the relationship between miRNAs or genes and GO terms or KEGG pathways, respectively.

### qRT-PCR to validate target genes and miRNA expression in mammary glands

From the cluster a, cluster b, and cluster c networks, the top 9, 10, and 5 target genes were selected according to degree score values, and 15 miRNAs were randomly screened from the transcriptome sequencing results. Primers for genes and miRNAs were designed by NCBI Primer-Blast [32] and miRprimer software [33], respectively. All primer sequences were provided in S10 Table. mRNA and miRNA quantification was performed according to the instructions of the One Step TB Green^®^ PrimeScript^™^ RT-PCR Kit (Perfect Real Time) (TaKaRa) and Mir-X^™^ miRNA qRT-PCR TB Green^™^ Kit (Clontech), respectively. PCR was performed on a LightCycler 96 (Roche) instrument. The reaction conditions were as follows: denaturation, 95 °C for 10 s; amplification, 40 cycles of 95 °C for 5 s and 60 °C for 20 s; and melting curve construction, 95 °C for 60 s, 55 °C for 30 s and 95 °C for 30 s. All experiments were repeated independently 3 times, and the results were calculated using the 2^-ΔΔCt^ method [34]. Tukey's honestly significant difference (HSD) test was used to test the significance of mRNA expression. The qRT-PCR results were visualized using GraphPad 7.0 (GraphPad Software, USA).

## Results

### Sequencing data and alignment analysis of small RNAs

After constructing libraries for the mammary gland tissue of 9 Laoshan dairy goats, we acquired 236833302 raw reads from sequencing. After quality control of the sequencing data, 11150238 reads of poor quality were filtered out, and 225683064 clean reads were retained (sequencing data statistics for each library are provided in S2 Table). By comparing the clean reads with the GtRNAdb, Rfam and Repbase databases, the numbers of rRNA, tRNA, snoRNA, snRNA, and repeat reads were obtained (Table 1). Ultimately, 20978810 ± 3843153, 20613062 ± 4463850, and 29444440 ± 5723011 unannotated reads (mean ± SE) at the LL, DP, and LG stages, respectively, were aligned with the genome (Table 1). Analysis of the unannotated reads aligned to the genome showed that the genomic alignment ratios of the reads from all 9 libraries were above 65% (S3 Table).

**Table 1:** ncRNA and repeat sequence annotation.

Sample ID	rRNA	tRNA	snoRNA	Repeat reads	snRNA	Unannotated	Total
LL	1142879±230692	72338±15193	81846±12167	107998±23940	4±1	20978810±3843153	22383874±4063836
DP	660829±307519	37392±8468	63955±9075	61913±21114	26±13	20613062±4463850	21437177±4793732
LG	1570043±601148	105702±16251	145769±24722	140662±64097	22±5	29444440±5723011	31406637±6389135

### Identification of known and novel miRNAs

**Fig 1:**
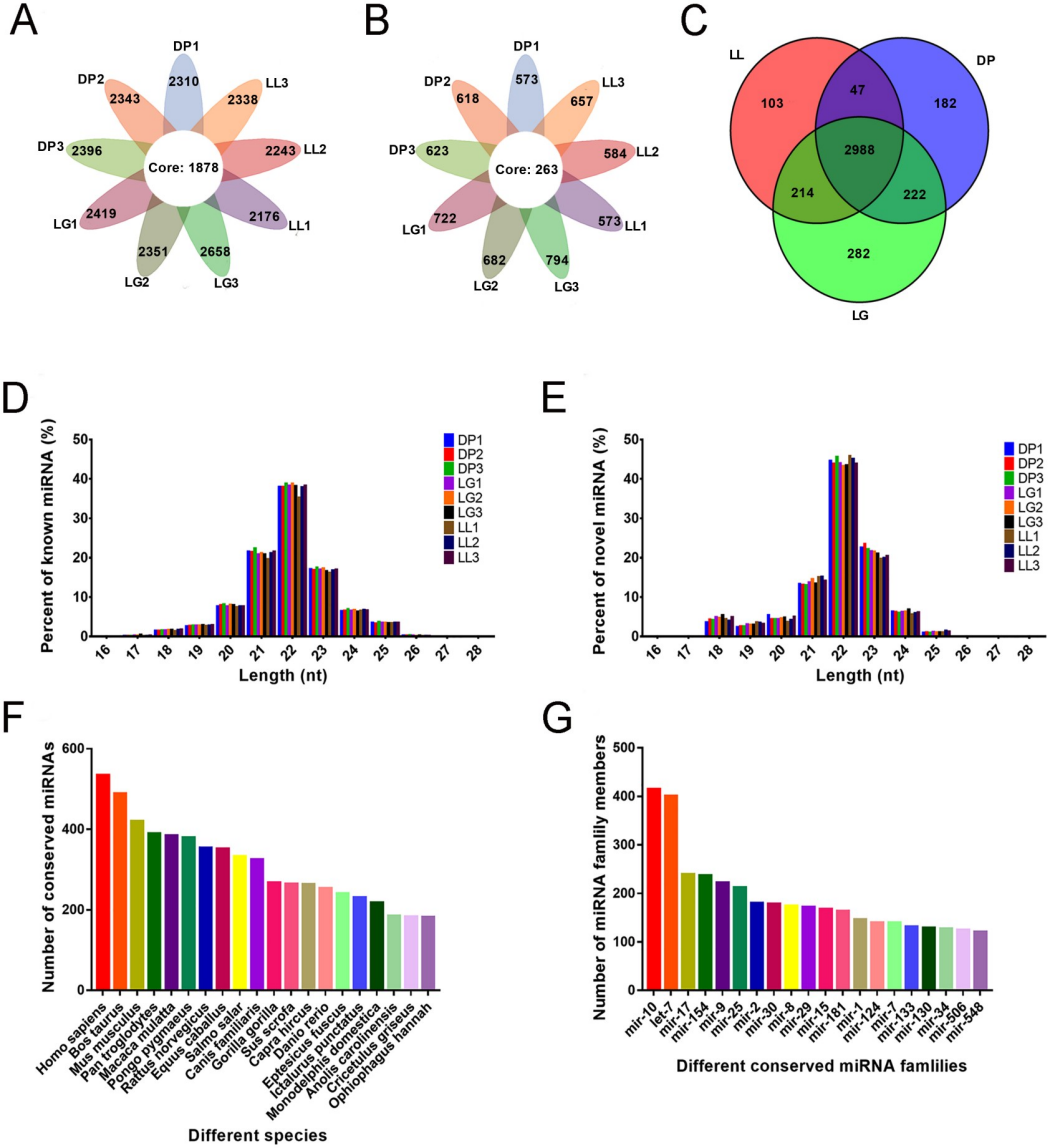
Identification and family analysis of known and novel miRNAs at different developmental stages. A-B present statistics of known and novel miRNAs identified from the 9 libraries. The core circle represents the total number of miRNAs in the 9 libraries. A shows the number of known miRNAs in the 9 libraries. B shows the number of novel miRNAs. C shows a Venn diagram of miRNAs at the three different stages. D-E show the distributions of the lengths of known and novel miRNAs. D shows the known miRNA length distribution, while E shows the novel miRNA length distribution. F shows the statistics of the number of all miRNAs in some species during the three stages. G shows the statistics of the number of miRNAs in different miRNA families.

As shown in Fig 1A and 1B, 4038 miRNAs were acquired from the 9 libraries, including 3129 known miRNAs and 909 newly predicted miRNAs. Among the known miRNAs, 1878 were expressed in all 9 libraries, and among the newly predicted miRNAs, 263 were expressed in all 9 libraries. We further determined miRNAs in each lactation period and identified 3352 miRNAs during the LL stage, 3439 miRNAs during the DP stage and 3706 miRNAs during the LG stage, among which 2988 miRNAs were expressed in all 3 lactation periods (Fig 1C). Analysis of the lengths of known and novel miRNAs showed that they were concentrated between 20 and 24 nt, with 22 nt miRNAs being the most abundant (Fig 1D and 1E). To further determine the miRNA expression pattern in the mammary gland tissue during the 3 different stages, the miRNAs were divided into 4 groups according to their TPM values (Table 2). In the high-expression group, the number of miRNAs at the DP stage was greater than that at the LL and LG stages, and in the medium-expression group, the number of miRNAs at the DP stage was the same as that at the LG stage and greater than that at the LL stage.

**Table 2:** Transcript expression profiles of the three lactation stages.

Different expression levels	LL	DP	LG
Highly expressed miRNAs (TPM ≥ 500)	135	196	150
Mediumly expressed miRNAs (500 > TPM ≥ 10)	725	787	787
Lowly expressed miRNAs (10 > TPM ≥ 1)	769	783	816
Ultralowly expressed miRNAs (1 > TPM)	2409	2272	2285

### miRNA family analysis

Through miRNA family analysis, we compared the sequence similarities of miRNAs among 104 species and identified 3316 miRNAs in 385 miRNA families. mir-9 and mir-10 were the most conserved, as they were matched in 84 species. The sequences of 24 miRNAs were conserved in more than 40 species. Quantification of the number of conserved miRNAs in different species showed that most miRNAs matched human miRNAs, with 534 miRNAs identified, followed by cattle and mice miRNAs, with 489 and 420 miRNAs identified, respectively. We found 263 conserved miRNAs in goats; the numbers of conserved miRNAs in different species are shown in Fig 1F. Quantification of miRNA numbers in different miRNA families showed that the mir-10 family had the most miRNAs, with 415, 6 miRNA families had more than 200 miRNAs and 22 families had more than 100 miRNAs. The numbers of conserved miRNAs in different miRNA families are shown in Fig 1G. Detailed results of the family analysis are included in S4 Table.

### Standardization of the sequencing data and identification of differentially expressed miRNAs

**Fig 2:**
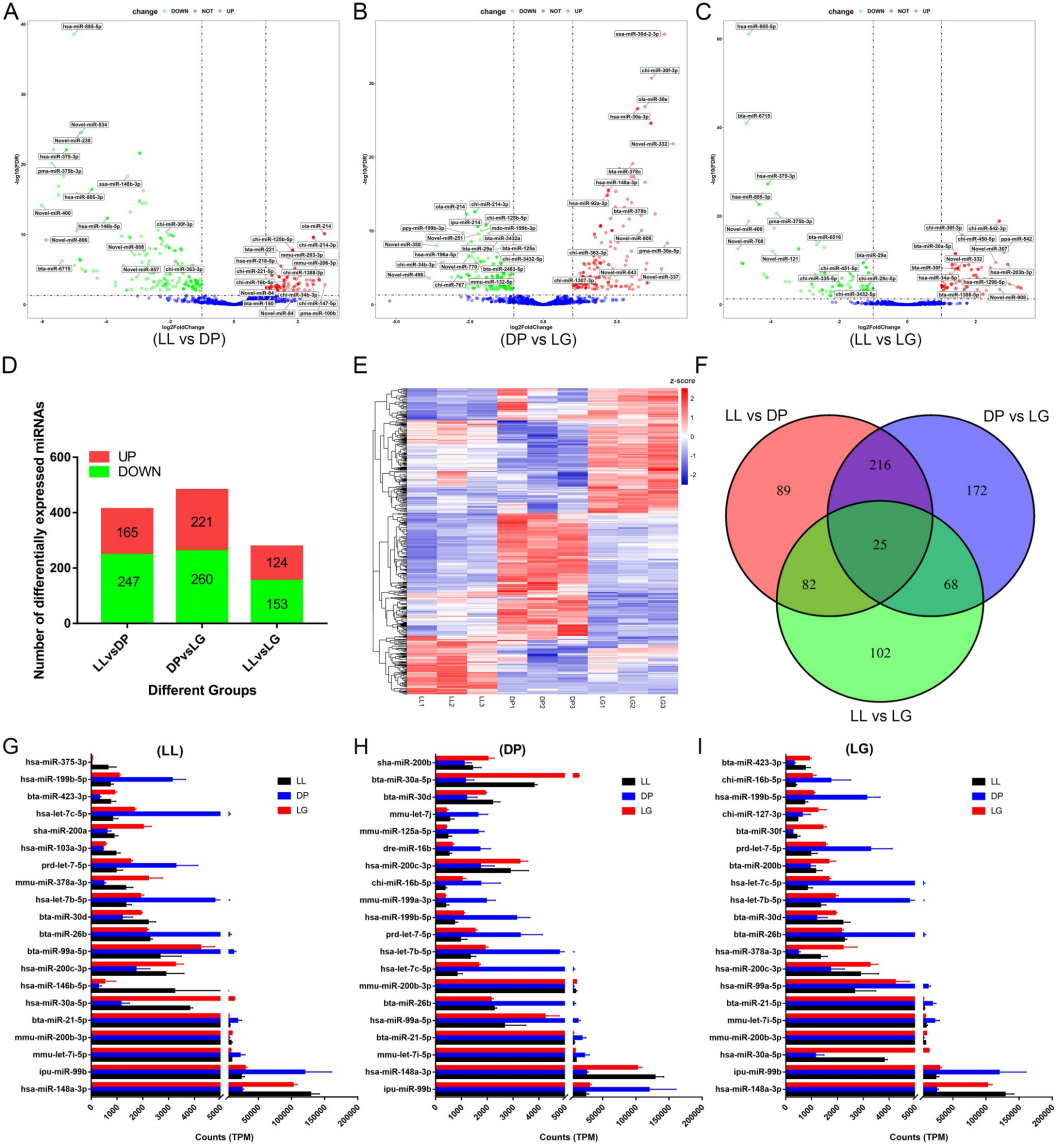
Identification of differentially expressed miRNAs. A-C show volcano plots of differentially expressed miRNAs among groups. A shows a volcano plot of miRNAs differentially expressed between the late lactation (LL) and dry period (DP) stages. B shows a volcano plot of miRNAs differentially expressed between the DP and late gestation (LG) stages. C shows a volcano plot of miRNAs differentially expressed between the LL and LG stages. D shows the number of upregulated and downregulated miRNAs in the three groups. E shows a heat map of the 754 differentially expressed miRNAs. F shows a Venn diagram of differentially expressed miRNAs among the three groups. G-I show the expression patterns of the 20 most differentially expressed miRNAs at the LL, DP, and LG stages, respectively.

Standardization of RNA-seq data helps accurately assess miRNA expression levels. We analyzed and standardized the 9 libraries for the LL, DP and LG stages using RUVSeq software and found that after standardization of the sequencing data, the expression levels in the samples from the same stage were more consistent (S1 Fig, where A and B present the data before standardization and C and D presented the data after standardization) and that the LL, DP and LG stages were distinguished by the first and second principal components of PCA (S1D Fig). We calculated the log2(TPM+1) of all samples, performed cluster analysis and found the samples in the same stage were clustered together, and there were some differences among the 3 stages (S1E Fig). The standardized data were imported into DESeq2 software, and the hypothesis of differential expression was tested for LL vs DP, DP vs LG and LL vs LG through the Wald test and Benjamini-Hochberg correction. We identified 754 differentially expressed miRNAs in the 3 groups (Fig 2). In the LL vs DP comparison, 185 miRNAs were upregulated and 247 miRNAs were downregulated. The differential expression of 16 of these miRNAs was higher than 32×, and the change in expression level was largest for novel-miR-400, at 64.7×. In the DP vs LG comparison, 221 miRNAs were upregulated and 260 miRNAs were downregulated. The upregulated miRNA novel-miR-332 showed the largest change, measuring 21.2×. In the LL vs LG comparison, 124 miRNAs were upregulated, 153 miRNAs were downregulated, and the downregulation of novel-miR-708 reached 31.2×. Detailed differential expression results are provided in S5 Table. According to the expression levels of the 20 most differentially expressed miRNAs at each developmental stage (Fig 2G–2I), 12 miRNAs (hsa-miR-148a-3p, ipu-miR-99b, bta-miR-30d, mmu-let-7i-5p, hsa-let-7b-5p, prd-let-7-5p, hsa-let-7c-5p, mmu-miR-200b-3p, hsa-miR-200c-3p, bta-miR-21-5p, bta-miR-26b, and hsa-miR-199b-5p) were expressed at high levels in mammary gland tissues at the three developmental stages (TPM ≥ 500).

### Expression pattern analysis of differentially expressed miRNAs

**Fig 3:**
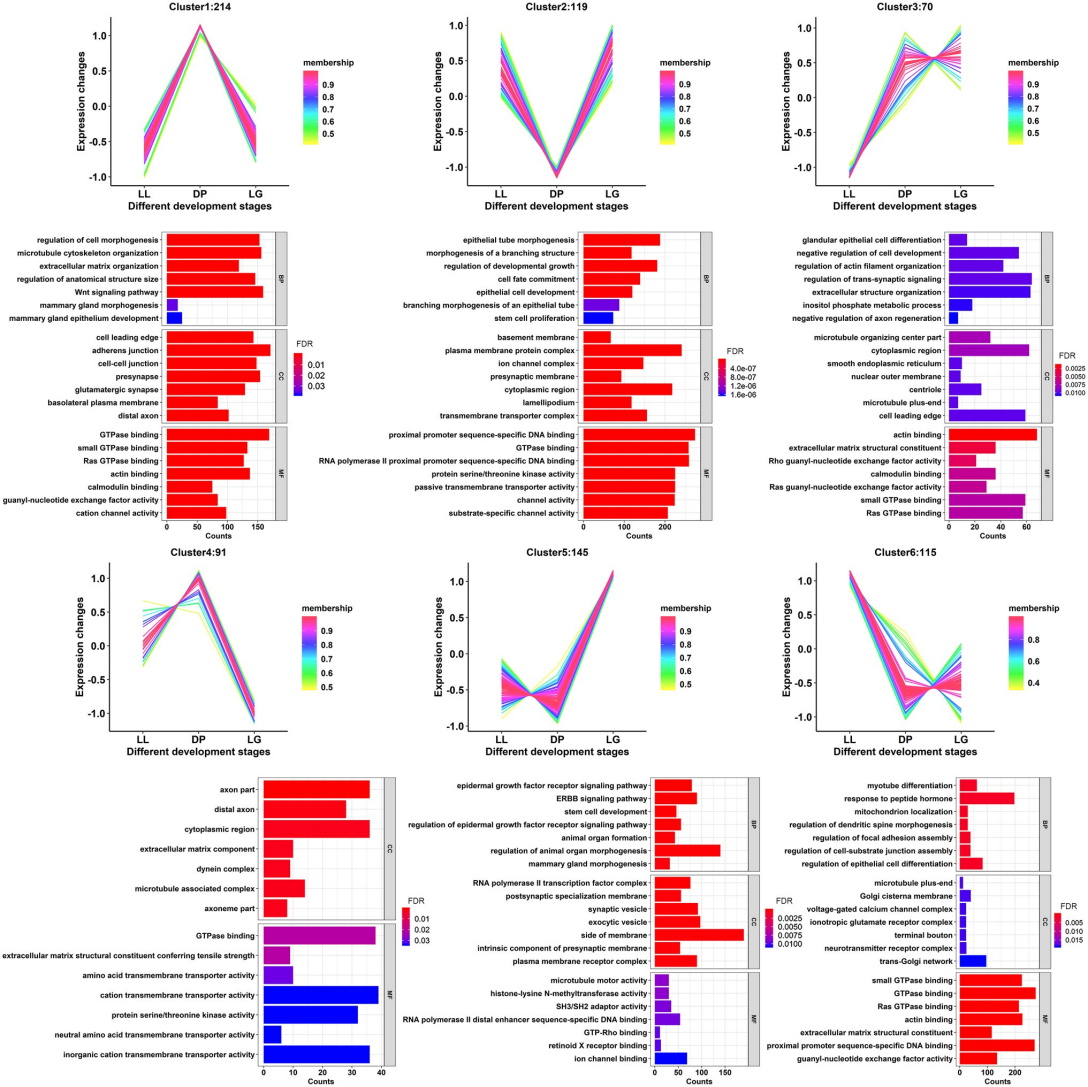
Expression pattern analysis of differentially expressed miRNAs. Clusters 1–6 show the cluster diagrams of all differentially expressed miRNA expression patterns and the GO enrichment analysis diagram of their target genes. The membership values indicate the degree to which data points belong to a cluster. The values of expression change indicate the expression level after normalizing the TPM values of miRNAs by the z-score method.

To further understand the expression patterns of the differentially expressed miRNAs during the 3 different stages, the miRNAs were clustered into 6 groups using the c-means method according to their expression levels (Fig 3). Additionally, the miRNA membership value in each cluster was calculated. Membership values reflect the degree to which data points belong to a cluster. The trends of miRNA expression in cluster 1 and cluster 4 were similar, with the expression levels increasing from the LL to DP stage and decreasing from the DP to LG stage. The expression levels of miRNAs in cluster 2 decreased from the LL to DP stage and increased from the DP to LG stage (detailed cluster information is provided in S6 Table). By analyzing the GO functions of target genes corresponding to each cluster of miRNAs, it was found that in cluster 1, target genes were annotated to regulation of cell morphogenesis (GO:0022604), mammary gland morphogenesis (GO:0060443), mammary gland epithelium development (GO:0061180) and other terms related to mammary gland morphogenesis. Target genes in cluster 2 were significantly annotated to cell fate commitment (GO:0045165), branching morphogenesis of an epithelial tube (GO:0048754) and other terms related to cell fate and tissue branch formation. Detailed GO analysis results are provided in S7 Table.

### GO and KEGG analyses of candidate target genes

**Fig 4:**
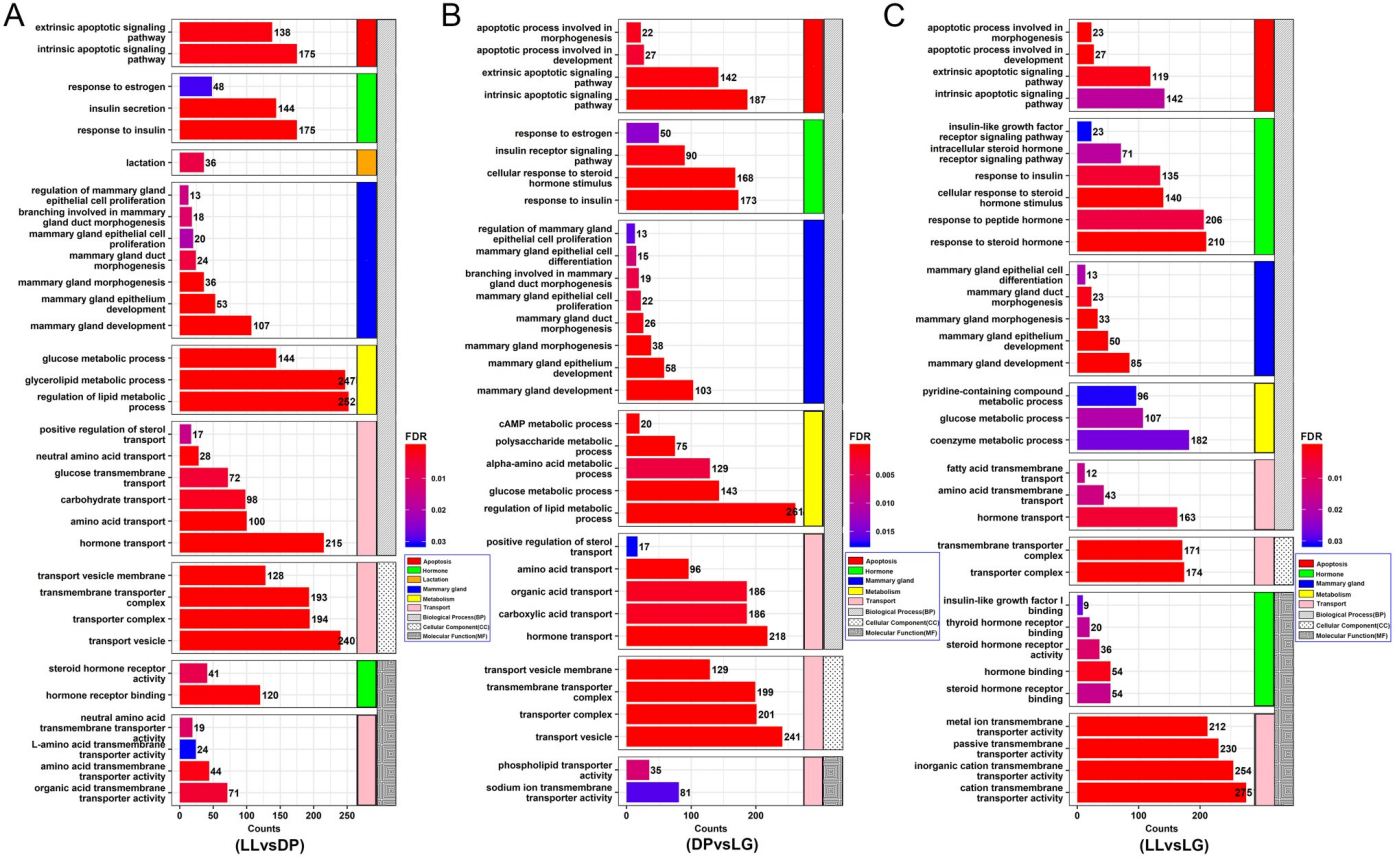
GO analysis of target genes corresponding to differentially expressed miRNAs. A-C show GO annotation maps of the target genes corresponding to differentially expressed miRNAs in different groups. The horizontal axis of the bar chart represents the number of target genes associated with each GO term, the vertical axis represents different GO terms, and the different colors of the bar chart represent FDR values.

**Fig 5:**
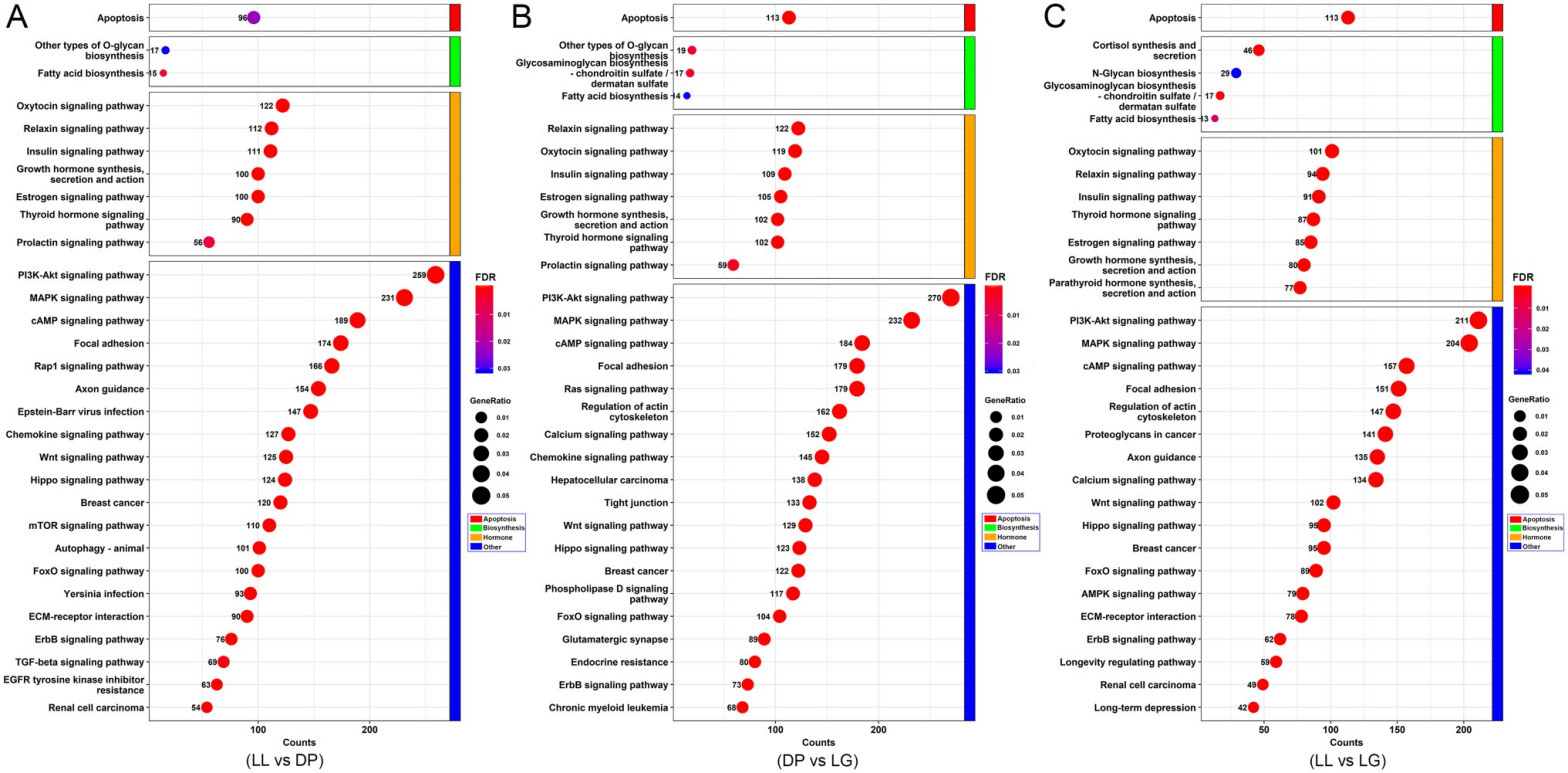
KEGG analysis of target genes corresponding to differentially expressed miRNAs. A-C show KEGG analysis maps of target genes corresponding to differentially expressed miRNAs in three groups. The horizontal axis of the bubble graph represents the number of target genes in each KEGG pathway, the vertical axis represents different channel names, the different colors of bubbles represent the FDR values, and the size of the bubbles represents the gene ratio value.

By arranging the GO annotation results (Fig 4A–4C), we identified a number of GO terms related to mammary gland growth, differentiation, apoptosis and lactation. Among them, apoptosis-related GO terms included intrinsic apoptotic signaling pathway (GO:0097193), extrinsic apoptotic signaling pathway (GO:0097191) and other biological processes. Hormone-related GO terms included response to estrogen (GO:0043627), insulin secretion (GO:0030073), cellular response to steroid hormone stimulus (GO:0071383) and other biological processes. In addition, mammary gland development and lactation are inseparable from the transport of substances. Some target genes were annotated as being closely related to positive regulation of sterol transport (GO:0032373), neutral amino acid transport (GO:0015804), glucose transmembrane transport (GO:1904659) and other processes. Substance metabolism is also crucial for mammary gland development. GO terms related to substance metabolism included cAMP metabolic process (GO:0046058), glucose metabolic process (GO:0006006), and regulation of lipid metabolic process (GO:0019216). Interestingly, mammary gland duct morphogenesis (GO:0060603), mammary gland morphogenesis (GO:0060443), mammary gland epithelium development (GO:0061180), mammary gland development (GO:0030879) and other mammary gland-related biological processes were screened out. The genes associated with these GO terms are coordinately expressed to support the functions and physiological processes of each stage of mammary gland development. According to the KEGG results (Fig 5A–5C), the target genes of miRNAs may be related to apoptosis (chx04210), fatty acid biosynthesis (chx00061), N-glycan biosynthesis (chx00510), the insulin signaling pathway (chx04910), the PI3K-Akt signaling pathway (chx04151), the MAPK signaling pathway (chx04010), the Wnt signaling pathway (chx04310), and the hippo signaling pathway (chx04392). These pathways also involve processes such as material anabolism, tissue development, cell proliferation, and apoptosis, which further indicates that the genes expressed at the three stages and their corresponding miRNAs may have regulatory effects on mammary gland development. Detailed GO analysis results are provided in S8 Table, and the KEGG analysis results are provided in S9 Table.

### Regulatory network of miRNAs and target genes

**Fig 6:**
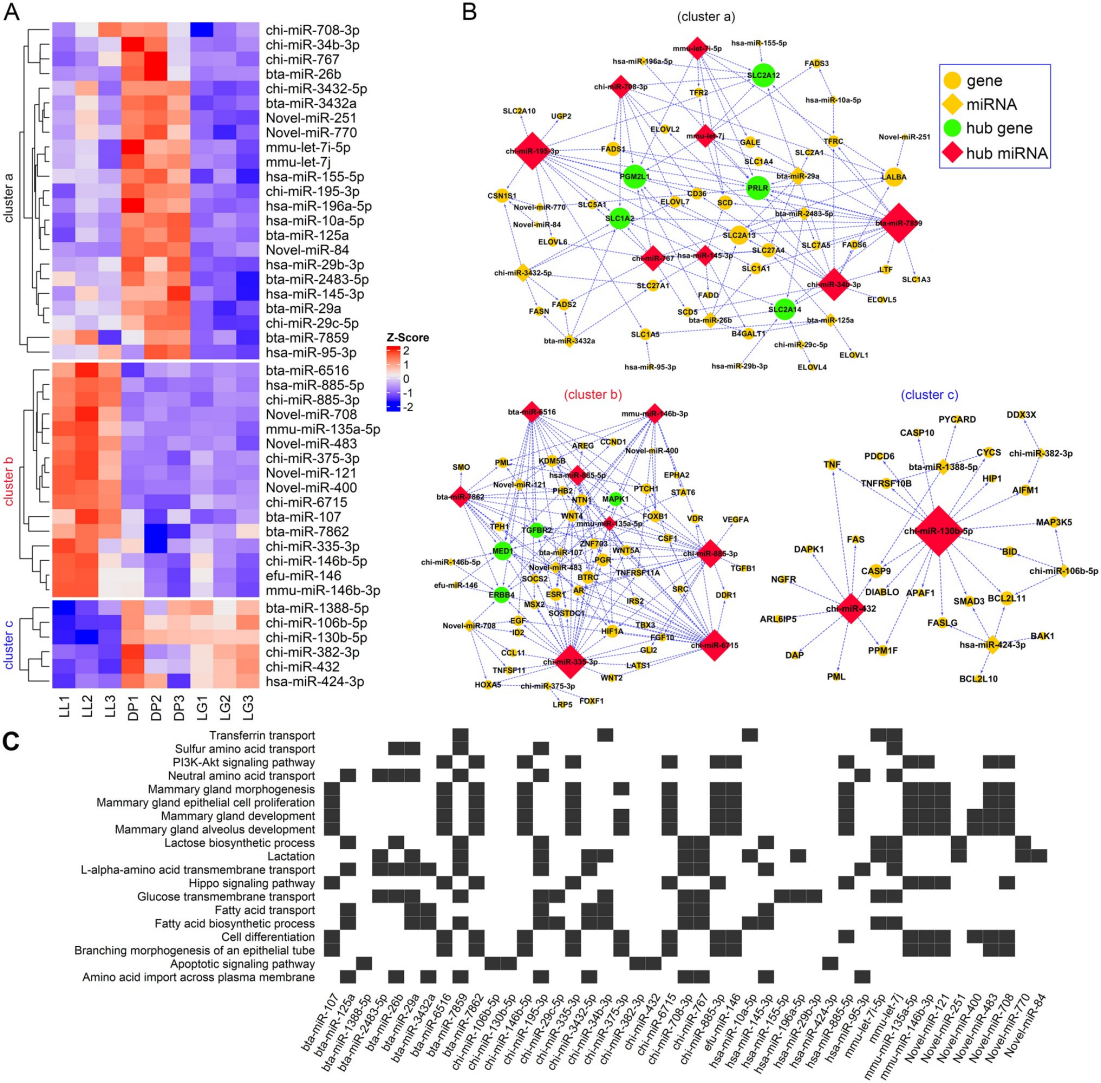
Network between differentially expressed miRNAs and their potential target genes. A shows a heat map of the expression profiles of 45 differentially expressed miRNAs at the three developmental stages. B shows a regulatory network of three miRNAs and potential target genes. The cluster a network contained 62 nodes and 123 regulatory relationships (edges), cluster b contained 63 nodes and 160 regulatory relationships, and cluster c contained 32 nodes and 39 regulatory relationships. The yellow circles and diamonds represent common genes and miRNAs, respectively. Green circles represent the hub target genes, and red diamonds represent the hub miRNAs. C shows a heat map of the relationship between GO terms or KEGG pathways and miRNAs.

Based on the GO and KEGG target gene annotation results, a total of 111 genes and 45 differentially expressed miRNAs were screened from 17 GO terms and 2 KEGG pathways to construct a targeted regulatory network (Fig 6A–6C). According to k-means clustering results for the expression of miRNAs, 45 miRNAs were divided into three groups (cluster a, cluster b, and cluster c). Cluster a contained 23 miRNAs. The expression of these miRNAs at the DP stage was higher than that at the LL and LG stages. Cluster b contained 16 miRNAs, whose expression level at the LL stage was higher than that at the DP and LG stages. Cluster c contained 6 miRNAs, which were more highly expressed at the DP and LG stages compared with the LL stage. At the same time, three regulatory networks of miRNAs and target genes were obtained. Cluster a was composed of 62 nodes and 123 edges. The network contained 8 core miRNAs and 5 core genes. The number of regulatory target genes for bta-miR-7859, chi-miR-195-3p, chi-miR-34b-3p and chi-miR-767 was greater than or equal to 9. According to bioinformatics analysis, bta-miR-7859 and chi-miR-195-3p can target and regulate the core genes *PRLR*, *PGM2L1*, *SLC2A12* and *SLC1A2*, which may participate in or affect lactose biosynthetic process, lactation, glucose transmembrane transport, fatty acid biosynthetic process, amino acid import across the plasma membrane and other biological processes related to milk regulation and milk component synthesis. Cluster b contained 63 nodes and 160 regulatory relationships as well as 8 hub miRNAs and 4 hub genes. chi-miR-335-3p, chi-miR-6715, and chi-miR-885-3p regulated more than 20 target genes and participated in the regulation of physiological processes such as mammary gland morphogenesis, mammary gland development, and cell differentiation. Cluster c contained 32 nodes and 39 regulatory relationships, as well as 2 hub miRNAs. The gene *CASP9* has a targeted regulatory relationship with chi-miR-432, chi-miR-130b-5p, and bta-miR-1388-5p. *BCL2L11* has a targeted regulatory relationship with chi-miR-130b-5p, hsa-miR-424-3p, and chi-miR-106b-5p. All six miRNAs in this network were predicted to be related to the apoptotic signaling pathway.

### Quantitative analysis of target gene expression in three regulatory networks

**Fig 7:**
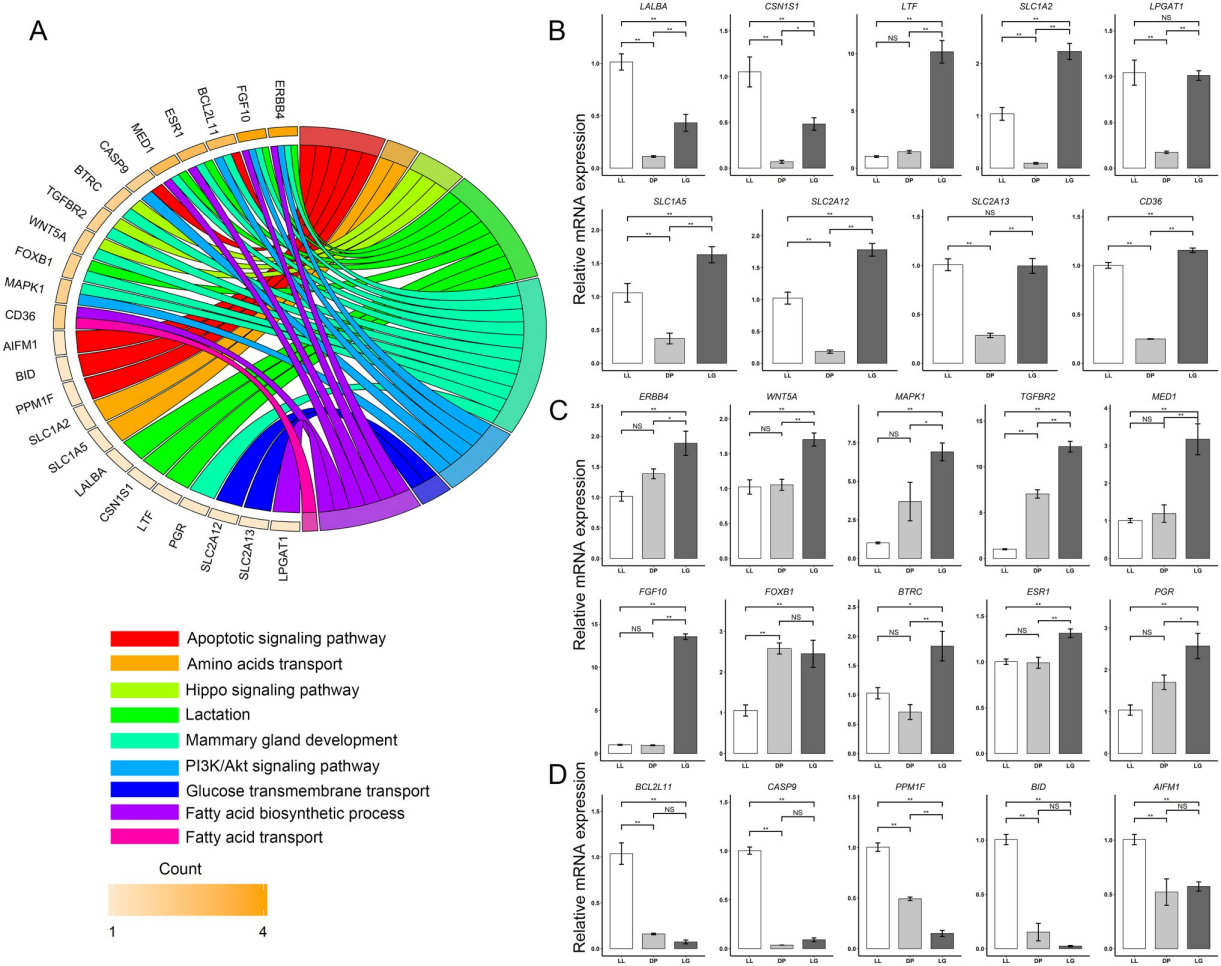
Quantitative analysis of target gene expression in three networks. A Chord plots of the relationships between target genes and GO terms or KEGG pathways. B shows a histogram of the relative expression of 9 genes in cluster a at the three developmental stages (LL, DP and LG). C shows a histogram of the relative expression of 9 genes in cluster b at the three developmental stages (LL, DP and LG). D shows a histogram of the relative expression of 9 genes in cluster c at the three developmental stages (LL, DP and LG). * indicates a statistically significant difference between groups (P < 0.05), ** indicates a highly significant difference between groups (P < 0.01), and NS (not significant) indicates that there is not a statistically significant difference between groups.

The results of quantitative analysis of target genes in the cluster a network (Fig 7A and 7B) showed that the expression levels of the lactation-related genes *LALBA* and *CSN1S1* at the LL stage were significantly higher than those at the DP and LG stages (P < 0.01). Among them, the expression level of *LALBA* at the LL stage was 9.09 ± 1.55 times and 2.34 ± 0.40 times higher than that at DP and LG stages. The expression level of *CSN1S1* at the LL stage was 15.18 ± 5.29 times and 2.18 ± 0.76 times higher than that at the DP and LG stages. There was no significant difference in *LTF* expression between the LL and DP stages, but the expression was significantly higher at the LG stage than at the LL and the DP stages by 7.14 ± 1.55 times and 10.02 ± 2.18 times, respectively. The amino acid transport-related genes *SLC1A2* and *SLC1A5* were expressed at lower levels at the DP stage, and the expression levels of *SLC1A2* at the LL and LG stages were significantly higher than the level at the DP stage by 11.17 ± 2.96 times and 24.02 ± 3.71 times, respectively, while the expression levels of *SLC1A5* at the LL and LG stages were significantly higher than the level at the DP stage by 2.85 ± 0.84 times and 4.38 ± 0.73 times, respectively. In addition, the expression levels of the glucose transmembrane transport-related gene *SLC2A12* at the LL and LG stages were significantly higher (by 5.60 ± 1.15 times and 9.74 ± 1.21 times, respectively) than the level at the DP stage, and the expression levels of *SLC2A13* at the LL and LG stages were significantly higher (by 3.53 ± 0.47 times and 3.49 ± 0.59 times, respectively) than the level at the DP stage. The expression of *LPGAT1*, a gene related to fatty acid biosynthesis, was significantly higher (by 6.00 ± 1.76 times and 5.82 ± 0.67 times, respectively) at the LL stage and DP stage than at the LG stage, and the expression of *CD36* was significantly higher (by 4.03±0.26 times and 4.65±0.21 times, respectively) at the LL and DP stages than at the LG stage. The results of quantitative analysis of target genes in the cluster b (Fig 7C) network showed that the expression of the mammary gland development-related genes *ERBB4*, *WNT5A*, *MAPK1*, *TGFBR2*, *MED1*, *FGF10*, *BTRC*, *ESR1*, and *PGR* was significantly higher at the LG stage than at the LL and DP stages. Among these genes, the hub gene *ERBB4* exhibited significantly higher expression at the LG stage than at the LL and DP stages, by 1.86 ± 0.44 times and 1.36 ± 0.32 times, respectively. The expression levels of the hub gene *MAPK1* at the LG stage were significantly higher than those at the LL and DP stages, specifically, 6.86±1.28 times and 1.87±0.35 times higher, respectively. The expression of the hub gene *TGFBR2* at the LG stage was significantly higher than that at the LL and the DP stages by 12.11 ± 1.30 times and 1.73 ± 0.19 times, respectively. The expression level of the core gene *MED1* at the LG stage was significantly higher than that at the LL and DP stages by 3.15 ± 0.91 times and 2.66 ± 0.77 times, respectively. In addition, *FGF10* was highly expressed at the LG stage, 13.45 ± 0.72 times and 14.17 ± 0.75 times higher than that at the LL and DPs stages, respectively. *FOXB1* expression was not significantly different between the DP and LG stages, but it was 2.44 ± 0.29 times and 2.32 ± 0.70 times higher at the DP stage and LG stage, respectively, than at the LL stage. Quantitative analysis of the target genes in the cluster c network (Fig 7D) showed that compared with those at the DP and LG stages, the apoptosis-related genes *BCL2L11*, *CASP9*, *PPM1F*, *BID*, and *AIFM1* were highly expressed at the LL stage. Specifically, the expression level of *BCL2L11* at the LL stage was 6.49 ± 1.64 times and 14.09 ± 3.56 times higher than that at the DP and LG stages, respectively, and the expression level of *CASP9* at the LL stage was 27.40 ± 2.23 times and 11.03 ± 0.90 times higher than that at the DP and LG stages, respectively.

### Validation of known and novel miRNAs using qRT-PCR

**Fig 8:**
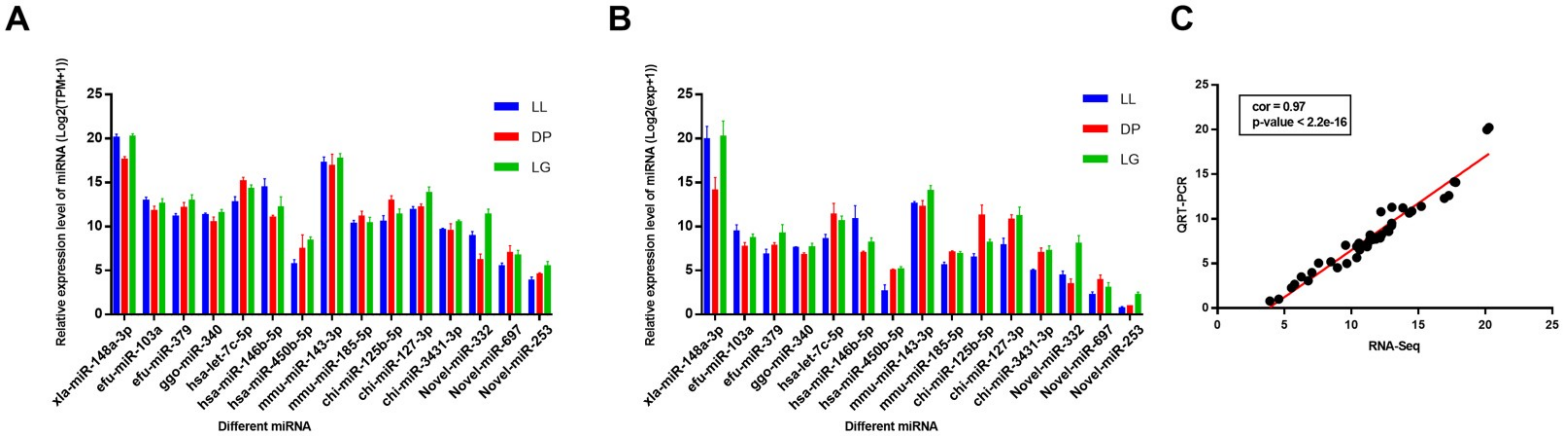
Validation of 15 randomly selected miRNAs by qRT-PCR. A shows the expression levels of 15 randomly selected miRNAs calculated by RNA-Seq. B shows the expression levels of 15 randomly selected miRNAs calculated by qRT-PCR. C Correlation between RNA-seq and qRT-PCR data. Fifteen miRNAs were randomly selected for validation.

The qRT-PCR results (Fig 8A and 8B) showed that the expression pattern of the 15 randomly selected miRNAs was consistent with the Illumina/Solexa sequencing results. Since the expression levels of the 15 randomly selected miRNAs varied substantially, to better present the trend of miRNA changes, we performed log2(exp+1) conversion of the expression levels and conducted correlation analysis between RNA-seq data and qRT-PCR data (Fig 8C) and found that the correlation (cor) value was 0.97 (P < 2.2e-16). This finding confirmed the reliability of the sequencing results.

## Discussion

During different stages of mammary gland development, mammary gland structure and physiological function change correspondingly, and these changes are influenced by various hormones, genes and regulatory factors. As important regulatory factors of gene expression, miRNAs play critical roles in many aspects, including metabolism, disease occurrence, mammary gland development and lactation regulation [35]. The purpose of this study was to map the expression profiles of miRNAs differentially expressed at the three stages of mammary gland development (LL, DP, and LG) through bioinformatic analysis in order to screen and identify miRNAs with potential regulatory effects on mammary gland tissue growth, differentiation, and lactation physiological processes. A total of 4038 miRNAs were identified at the three stages, including 3129 known miRNAs and 909 newly predicted miRNAs. Not all of these miRNAs were expressed at all stages; in fact, only 2988 miRNAs were commonly expressed among the three stages (Fig 1C), which also indicates that the expression of many miRNAs is time-specific [36]. In addition, the miRNAs that we identified were mostly distributed in the 20–24 nt length range, with the greatest abundance of 22 nt miRNAs (Fig 1D and 1E). This finding is consistent with the typical length distribution of miRNAs found in most mammals, for example, sheep [37], pig [38], and mouse [39]. A statistical analysis of the identified miRNAs in various species revealed that 534 miRNAs were annotated in and related to humans, 489 miRNAs were related to cattle, and 263 miRNAs were related to goats, with fewer miRNAs annotated to goats than to humans and cattle (Fig 1F). On the one hand, this pattern reflects the limited annotation information available for goat-related miRNAs in the database and shows that it is necessary to further identify and functionally annotate such miRNAs. According to family analysis, the largest number of miRNAs was annotated to the mir-10 family, at 415, followed by let-7 and mir-17, with 401 and 239 miRNAs, respectively (Fig 1G). The mir-10 family is known to be highly conserved and is one of the most widely distributed microRNAs in animals [40]. Moreover, the conserved mir-10 family reportedly inhibits the proliferation of ovarian granulosa cells and induces apoptosis [41]. As the first miRNA to be found in *C*. *elegans*, let-7 and its family members are highly conserved throughout the species [42]. Studies have reported the sequences of high-abundance let-7 family members detected during the DP and peak lactation period [43]. Our previous research also showed that the number of let-7 family members in miRNA libraries for early lactation and LL stages is greater than 200 [22]. In addition, let-7b can regulate the growth hormone receptor (GHR) gene, affecting skeletal muscle growth and fat deposition [44]. These data indicate that miRNAs in the miR-10 family and let-7 family are regulators of basic biological processes and may have regulatory effects on mammary gland development and lactation.

There were significant differences in the expression of miRNAs in mammary gland tissue at different developmental stages. A total of 754 differentially expressed miRNAs were screened at the three developmental stages (Fig 2), and 20 miRNAs with high expression levels (TPM ≥ 500) were identified in each stage, among which miR-148a-3p, miR-99b, miR-21-5p and miR-30d were highly expressed not only in goat mammary gland tissue but also in human mammary cells [45]. We found that the expression levels of miR-148a-3p and miR-30d at the DP stage were significantly lower than those at the LL and LG stages and the expression levels at the LL stage were the highest. miR-148a-3p overexpression can induce apoptosis of epithelial cells [46] and regulates cell proliferation, differentiation, and epithelial-mesenchymal transitions [47]. miR-30d can also inhibit the proliferation of colon cancer cells and promote their apoptosis [48]. These miRNAs may be associated with the apoptosis of mammary gland tissues during the LL stage. However, the expression of miR-99b at the DP was significantly higher than that at the LL and LG stages. miR-99b can inhibit the expression of IGF-1R, thereby affecting cell proliferation [49]. This suggests miR-99b may be involved in the mammary gland tissue remodeling process during the DP stage. In addition, we found that let-7 family members such as let-7-5p, let-7b-5p, let-7c-5p and let-7i-5p had higher expression in mammary gland tissues at the three developmental stages, similar to the results found in other animals such as cattle [50] and mice [51]. This again suggests that the let-7 family may be critical for mammary gland development. The expression levels of miR-200c-3p and miR-200b-3p at the DP were significantly lower than those at the LL and LG stages, and the expression levels were highest at the LG stage. Overexpression of miR-200b or miR-200c in vitro can cause the appearance of LY2 human breast cancer cells to change from slender/fibroblast-like to cobblestone-like [52]. The above results suggest that these differentially expressed miRNAs may play an important role in mammary gland development, and multiple miRNAs may cooperate to participate in the physiological regulation of mammary gland development and lactation.

miRNAs play an important role in energy metabolism, including glucose and lipid metabolism and amino acid biosynthesis [53]. We found that miRNAs such as bta-miR-7859, chi-miR-195-3p, chi-miR-34b-3p and chi-miR-708-3p in cluster a have potential regulatory effects on the synthesis of milk components (Fig 6). The transport of amino acids, glucose, fatty acids and other basic substances is an important part of the synthesis of milk components such as protein, fat and lactose. The hub miRNA chi-miR-195-3p has a targeted regulatory relationship with *SLC1A1*, *SLC1A2*, *SLC1A5*, *SLC5A1*, *SLC2A10*, *SLC2A12*, *SLC2A13* and *CD36*. Both *SLC1A1* and *SLC1A2* can encode proteins with the ability to transport glutamate, and they play an important role in glutamate transport across the plasma membrane, while the SLC1A5 gene can encode a sodium-dependent neutral amino acid transporter [54]. The *SLC5A1* gene encodes a member of the sodium-dependent glucose transporter (SGLT) family, and the encoded integral membrane protein is the main mediator of dietary glucose and galactose intake from the intestinal cavity [55]. *SLC2A10*, *SLC2A12*, and *SLC2A13* are also associated with glucose transport [56]. In addition, *CD36* encodes important fatty acid transferases that not only are involved in fatty acid transport and lipid metabolism but also serve as potential prognostic biomarkers for cancer [57]. Our results showed that the expression levels of *SLC1A2*, *SLC1A5*, *SLC2A12*, *SLC2A13*, and *CD36* at the DP stage were significantly lower than those at the LL and LG stages, and the expression was highest at the LG stage (Fig 7B). This finding suggests that these genes play an important role in the synthesis of sugars, fats and proteins in milk during the LL and LG stages. The *LALBA* gene encodes a principal protein in milk, called alpha-lactalbumin, which regulates the production of lactose in the milk of almost all mammals [58]. Our results showed that it was identified as a hub gene regulated by chi-miR-34b-3p, chi-miR-708-3p, chi-miR-767, mmu-let-7i-5p, novel-miR-770, and novel-miR-251. miR-34b-3p and let-7i-5p can affect the proliferation and differentiation of adipocytes and affect the deposition of fat in animals [59]. miR-708-3p and miR-767 are associated with cancer cell proliferation [60, 61]. Interestingly, chi-miR-34b-3p expression was significantly higher at the DP than at the LL and LG stages, and *LALBA* expression was significantly lower at the DP than at the LL and LG stages. This result implies that the expression changes of chi-miR-34b-3p may impact changes in *LALBA* expression. In addition, we also identified the miRNAs chi-miR-195-3p, chi-miR-3432-5p, novel-miR-84, and novel-miR-770, which are related to the potential regulation of *CSN1S1*. We found that the expression patterns of *CSN1S1* and *LALBA* were similar across the three developmental stages, i.e., significantly lower at the DP stage than at the LL and LG stages and significantly higher at the LL stage than at the LG stage (Fig 7B). This result may be because the composition of milk at the LL stage is more similar to that of mature milk while that at the LL stage is more similar to that of colostrum. Alessandra et al. also found that the expression of *CSN1S1* and *LALBA* in mature milk was significantly higher than that in colostrum [62]. In addition, chi-miR-34b-3p and bta-miR-7859 may participate in the immune regulation of the mammary gland in different developmental stages. Both miRNAs can regulate the *LTF* gene, a member of the transferrin family with protein products existing in neutrophil secondary particles. The protein is the main iron binding protein in milk and human body secretions, and its antimicrobial activity makes it an important part of the nonspecific immune system [63, 64]. Our results showed that the expression level of *LTF* at LG stage was significantly higher than that at the LL and DP stages, which is closely related to the higher levels of *LTF* in colostrum, thus directly and indirectly protecting the newborn from bacterial and other microbial infections.

The influence of miRNAs on mammary gland tissue remodeling, growth and differentiation has long been a topic of interest. Our results indicate that miRNAs in cluster b have potential regulatory effects on mammary gland development, proliferation and differentiation. We found that the three hub miRNAs chi-miR-335-3p, chi-miR-6715, and bta-miR-6516 can target and regulate the four hub genes *ERBB4*, *MED1*, *TGFBR2* and *MAPK1*. *ERBB4* is a member of the tyrosine protein kinase family and epidermal growth factor receptor subfamily; it plays an important role in the maintenance of mammary gland bubbles and lactation and promotes the development of murine and human mammary epithelial cells in cell culture [65]. *MED1* encodes a coactivator and is involved in the transcriptional regulation of almost all RNA polymerase II-dependent genes. The mediator subunits MED1 and MED24 jointly promote the development of the adolescent mammary gland [66]. *TGFBR2* knockout mice develop structural degenerative lesions of mammary gland ducts and lobules, which aggravate mammary cell apoptosis [67]. Tissue remodeling is an important physiological process in mammary gland development, and *STAT3* and *MAPK* play an important role in mammary gland remodeling [68]. We found that the expression levels of these four genes at the LG stage were higher than those at the LL and DP stages (Fig 7C). These results further illustrate that the four genes may play an important role in promoting the physiological processes of mammary epithelial cell proliferation and differentiation, mammary gland acinar development, and formation of branch structure at the LG stage. Our results also showed that the hub miRNAs chi-miR-335-3p, chi-miR-6715, bta-miR-6516, chi-miR-885-3p, bta-miR-7862, mmu-miR-146b-3p, and mmu-miR-135a-5p may regulate the PI3K-Akt signaling pathway and hippo signaling pathway (Fig 6C). miR-335 can coordinate cell proliferation, migration and differentiation in human mesenchymal stem cells [69] and participates in the hippo signaling pathway to affect the growth and invasion of lung cancer [70]. The hippo signaling pathway was originally found to control the size of *Drosophila* organ, and its core structure is conserved in mammals [71]. miR-885, miR-146b-3p, and miR-135a-5p have also been reported to have an effect on cell growth by regulating the hippo signaling pathway [72–74]. However, there are few reports on the effects of chi-miR-6715, bta-miR-6516, and bta-miR-7862 on mammary gland growth and differentiation. In addition, novel-miR-708, novel-miR-483, novel-miR-121, novel-miR-400 and other newly screened miRNAs have also been identified as related to mammary gland development. We found that novel-miR-708 can target and regulate *TNFSF11*, *EGF*, *HOXA5* and other genes (Fig 6B). TNFSF11 is a member of the tumor necrosis factor superfamily, which is downregulated and leads to defects in the early differentiation of T and B lymphocytes in mice and affects the formation of acini and branching structures in the mammary gland during pregnancy [75]. EGF stimulates cell growth and differentiation by binding to its receptor EGFR [76]. In summary, the miRNAs and potential target genes identified in cluster b may be involved in the regulation of mammary gland development and differentiation.

In cluster c, we also identified miRNAs associated with apoptosis in the mammary gland (Fig 6B). In this network, chi-miR-130b-5p, chi-miR-432, bta-miR-1388-5p, hsa-miR-424-3p, chi-miR-106b-5p, and chi-miR-382-3p participate in the regulation of the apoptotic signaling pathway and may be beneficial to the proliferation of mammary epithelium. Increased expression of miR-130b promotes cell proliferation, stimulates G0/G1 phase cells to enter S phase, inhibits apoptosis, and promotes tumor invasion and metastasis [77]. We found that chi-miR-130b-5p can target and regulate the apoptosis-related genes *CASP9*, *BCL2L11*, *BID*, *PPM1F* and *APAF1*. The expression level of chi-miR-130b-5p is lower at the LL stage than at the DP and LG stages, which is contrary to the expression pattern of the five apoptosis-related genes (Fig 7D). This suggests that chi-miR-130b-5p may inhibit apoptosis and promote mammary gland development and mammary cell proliferation. chi-miR-106b-5p inhibits apoptosis, and it has a targeted regulatory relationship with *MAP3K5*, *BCL2L11*, and *BID*. The *MAP3K5*-encoded protein belongs to the MAP3K family, which is an important part of the MAP kinase signal transduction pathway and can induce apoptosis by activating JNK and p38 [78]. Overexpression of miR-106b-5p in glioma cells can promote significant cell proliferation and mediate apoptosis of glioma cells by targeting CASP8 [79]. However, in contrast to our findings, Nan et al. reported that miR-432 inactivated the Wnt/β-catenin pathway by simultaneously inhibiting the expression of *LRP6*, *TRIM29*, and *Pygo2*, thereby inhibiting the proliferation of human hepatocellular carcinoma [80]. This may be because miRNA-432 plays a different role in different tissue cells and has tissue specificity. In addition, its function may also be affected by various hormones, cytokines and other factors in the body. The above results indicate that these miRNAs in cluster c may play an important role in mammary cell apoptosis.

## Conclusions

In summary, in this study, we constructed miRNA regulatory networks involved in mammary gland development and lactation regulation through miRNA sequencing during three distinct mammary gland developmental stages. Using target gene prediction and functional annotation analyses, we identified core regulatory elements of miRNAs. This study provides important information about existing and novel miRNAs that may govern goat mammary development and lactation and hence are likely to serve as valuable molecular markers in dairy goat selection.

## Supporting information

S1 Fig Quality control and standardization of miRNA expression. A-B show the analyses before data standardization, where A represents the relative logarithmic expression analysis of all samples and B represents the principal component analysis. C-D show the analyses of all samples after data standardization, where C represents the relative logarithmic expression analysis of all samples and D represents the principal component analysis. E shows a heatmap obtained by cluster analysis of all samples according to the correlation of expression levels.
(TIF)

S1 Table Mammary gland developmental stage, age, parity and body size of Laoshan dairy goats used for microRNA profiles analysis. (XLSX)

S2 Table Statistics for the number of sequencing reads in the nine libraries. (XLSX)

S3 Table Summary of sequencing read alignment to the goat reference genome. (XLSX)

S4 Table Analysis and statistics of miRNA families. (XLSX)

S5 Table Differentially expressed miRNA results between the three groups. (XLSX)

S6 Table Clustering analysis results of differentially expressed miRNAs. (XLSX)

S7 Table GO annotation results for target genes of six clusters of differentially expressed miRNAs. (XLSX)

S8 Table GO annotation results for target genes of three groups of differentially expressed miRNAs. (XLSX)

S9 Table KEGG results for target genes of three groups of differentially expressed miRNAs. (XLSX)

S10 Table Primer sequences of miRNAs for qRT-PCR. (XLSX)
