# Supplementary figures and images for: Characterization of microRNA profiles in the mammary gland tissue of dairy goats at the late lactation, dry period and late gestation stages
Source: PLoS One. 2020 Jun 8;15(6):e0234427. doi: 10.1371/journal.pone.0234427 (PMC7279595; doi:10.1371/journal.pone.0234427)

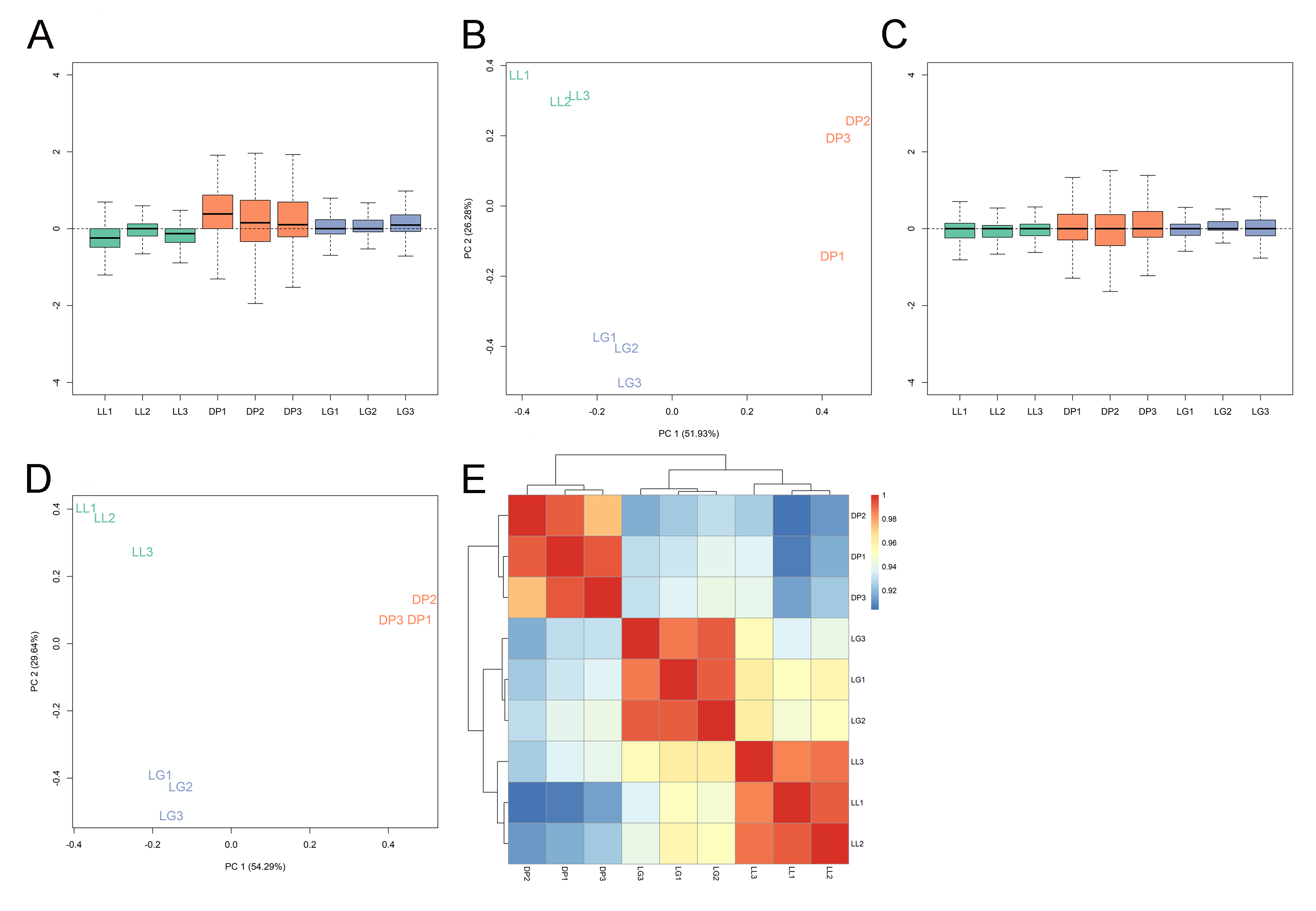

Supplement: S1 Fig — A-B show the analyses before data standardization, where A represents the relative logarithmic expression analysis of all samples and B represents the principal component analysis. C-D show the analyses of all samples after data standardization, where C represents the relative logarithmic expression analysis of all samples and D represents the principal component analysis. E shows a heatmap obtained by cluster analysis of all samples according to the correlation of expression levels. (TIF) [file pone.0234427.s001.tif]
